# Plasticity of lung cancer stem-like cells is regulated by the transcription factor *HOXA5* that is induced by oxidative stress

**DOI:** 10.18632/oncotarget.10571

**Published:** 2016-07-13

**Authors:** Hiroshi Saijo, Yoshihiko Hirohashi, Toshihiko Torigoe, Ryota Horibe, Akari Takaya, Aiko Murai, Terufumi Kubo, Toshimitsu Kajiwara, Tsutomu Tanaka, Yosuke Shionoya, Eri Yamamoto, Reo Maruyama, Munehide Nakatsugawa, Takayuki Kanaseki, Tomohide Tsukahara, Yasuaki Tamura, Yasushi Sasaki, Takashi Tokino, Hiromu Suzuki, Toru Kondo, Hiroki Takahashi, Noriyuki Sato

**Affiliations:** ^1^ Department of Pathology, Sapporo Medical University School of Medicine, Sapporo, 060-8556, Japan; ^2^ Department of Respiratory Medicine and Allergology, Sapporo Medical University School of Medicine, Sapporo, 060-8556, Japan; ^3^ Department of Molecular Biology, Sapporo Medical University School of Medicine, Sapporo, 060-8556, Japan; ^4^ Department of Molecular Therapeutics, Center for Food and Medical Innovation, Hokkaido University, Sapporo, 060-8638, Japan; ^5^ Department of Medical Genome Sciences, Research Institute for Frontier Medicine, Sapporo Medical University School of Medicine, Sapporo, 060-8556, Japan; ^6^ Division of Stem Cell Biology, Institute for Genetic Medicine, Hokkaido University, Sapporo, 060-8638, Japan

**Keywords:** cancer stem cell, plasticity, lung cancer, SOX2, HOXA5

## Abstract

Cancer stem-like cells (CSCs)/cancer-initiating cells (CICs) are reasonable targets for cancer therapy. However, recent studies have revealed that some non-CSCs/CICs have plastic ability and can dedifferentiate into CSCs/CICs. Therefore, an understanding of the molecular mechanisms that control the plasticity is essential to achieve CSC/CIC-targeting therapy. In this study, we analyzed the plasticity of lung cancer cells and found that lung non-CSCs/CICs can dedifferentiate into CSCs/CICs in accordance with the expression of stem cell transcription factor *SOX2*. *SOX2* expression was induced by the transcription factor *HOXA5*. Oxidative stress repressed the expression of *HDAC8* and then induced histone 3 acetylation and increased the expression of *HOXA5* and *SOX2*. These findings indicate that lung cancer cells have plasticity under a condition of oxidative stress and that *HOAX5* has a critical role in dedifferentiation.

## INTRODUCTION

Advanced lung cancer is a lethal disease and very large numbers of patients die annually worldwide [1, 2]. In addition to conventional lung cancer treatments including surgery, radiation and chemotherapy, molecular targeting drugs have recently been developed for treatment of lung cancer and have shown some prognostic advantages [3, 4]; however, the merits for patients remain limited [5, 6]. Elucidation of the biological properties of lung cancer cells is essential to find a cure for lung cancer.

Cancer stem-like cells (CSCs)/cancer-initiating cells (CICs) are thought to be major causes of cancer recurrence, distant metastasis and treatment resistance [7]. The idea of CSCs/CICs has a long and winding story [8], but the first evidence for the existence of CSCs/CICs was obtained for leukemia stem cells [9, 10], and the concept of ‘cancer stem cell’ became important in the cancer research field. CSCs/CICs in a solid tumor were first isolated from breast carcinoma with the combination of CD44 and CD24 expression [11]. CSCs/CICs are thought to be located at the top of a hierarchical differentiation model and maintain themselves by self-renewal. CSCs/CICs produce differentiated non-CSCs/CICs at the same time to form a heterogenic cancer population [12]. According to a cancer stem cell hypothesis, only a fraction of cancer cells have strong clonogenic ability and give rise to a predictable hierarchical model of tumor growth [13]. On the other hand, some groups have recently demonstrated that non-CSCs/CICs can acquire stem-like properties in breast cancer, and those groups obtained proof that cell plasticity is maintained without genetic manipulation and that any subpopulation of cells will return to equilibrium phenotypic proportions over time [14–16]. Plasticity of non-CSC/CIC in colon cancers has also been described [17]. Lung cancer is a highly metastatic disease and treatments often fail due to recurrence, suggesting that CSCs/CICs might play a role in clinical causes. However, plasticity of lung cancer cells has not been determined yet.

Previously, we showed that lung CSCs/CICs can be isolated as side population (SP) cells and that the transcription factor *SOX2* is expressed in lung CSCs/CICs [18]. *SOX2* has an essential role in the maintenance of lung CSCs/CICs. In this study, we investigated the plasticity of lung CSCs/CICs by using *SOX2* as a lung CSCs/CICs marker and we found a novel mechanism of dedifferentiation of lung cancer cells.

## RESULTS

### Differentiated lung cancer cells dedifferentiate into cancer stem-like cells

**Figure 1:**
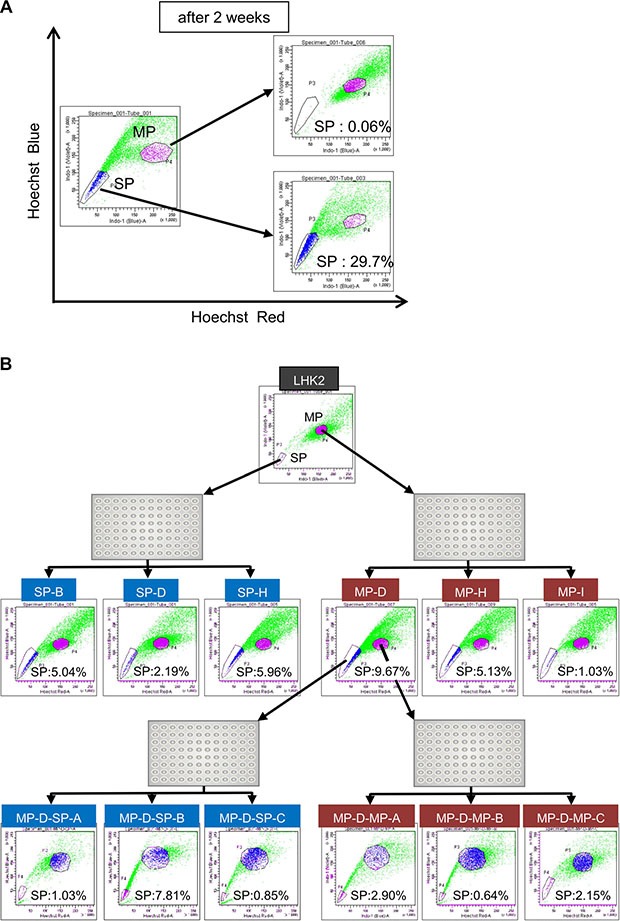
Differentiated non-CSCs/CICs dedifferentiate into CSCs/CICs (**A**) SP assay of LHK2 cells. The percentages represent ratios of SP cells and MP cells. Sorted SP cells and MP cells were cultured in DMEM supplemented with 10% FBS for 2 weeks and analyzed by the SP assay again. (**B**) SP assay of LHK2 SP clone cells and MP clone cells, and second generation of SP clone cells and MP clone cells derived from MP-D clone cells. The percentage represents ratio of SP cells.

In a previous study, we succeeded in isolating lung CSCs/CICs from the lung adenocarcinoma cell line LHK2 as side population (SP) cells [18]. In the present study, we analyzed the self-renewal and differentiation abilities of LHK2 SP cells and main population (MP) cells. SP cells showed higher tumor-initiating ability as described previously [18], and SP cell showed higher expressions of stem cell-related genes including *SOX2, ALDH1A1, KLF4* and *NANOG* (Supplementary Figure S1), indicating that SP cells are enriched with CSCs/CICs. Isolated SP cells and MP cells derived from LHK2 cells were cultured *in vitro* for 2 weeks, and then the cultured SP cells and MP cells were re-analyzed (Figure 1A). Cultured SP cells included a large percentage of SP cells (29.7%). Furthermore, some of the cultured SP cells had differentiated into MP cells, indicating that SP cells have both self-renew ability and differentiation ability. Interestingly, the proportion of SP cells in cultured MP cells was only 0.06% (Figure 1A). For detailed analysis, we investigated the differentiation status at the single cell level. Single cells were sorted from both SP cells and MP cells and cultured for more than one month until clone cells show stable growth. Several clones were established from both SP cells and MP cells, and clone cells were re-analyzed by an SP assay. Clones derived from SP cells were positive for SP cells (SP rates were 5.04% for SP clone B, 2.19% for SP clone D and 5.96% for SP clone H.) (Figure 1B). Interestingly, clones derived from MP cells were also positive for SP cells (SP rates were 9.67% for MP clone D, 5.13% for MP clone H and 1.03% for MP clone I.). Furthermore, we re-established MP clones and SP clones from one MP clone cells (MP-D). Both SP clones and MP clones derived from MP-D clone cells were positive for SP cells (Figure 1B). To confirm the phenomenon, we performed similar single cell sorting analysis using lung squamous cell carcinoma cell line, Sq-1. Both SP clone cells and MP clone cells showed positive for SP cells (Supplementary Figure S2). These results indicated that lung differentiated MP cells can dedifferentiate into SP cells.

### *SOX2* expression and stemness were regulated by class I *HDAC*

**Figure 2:**
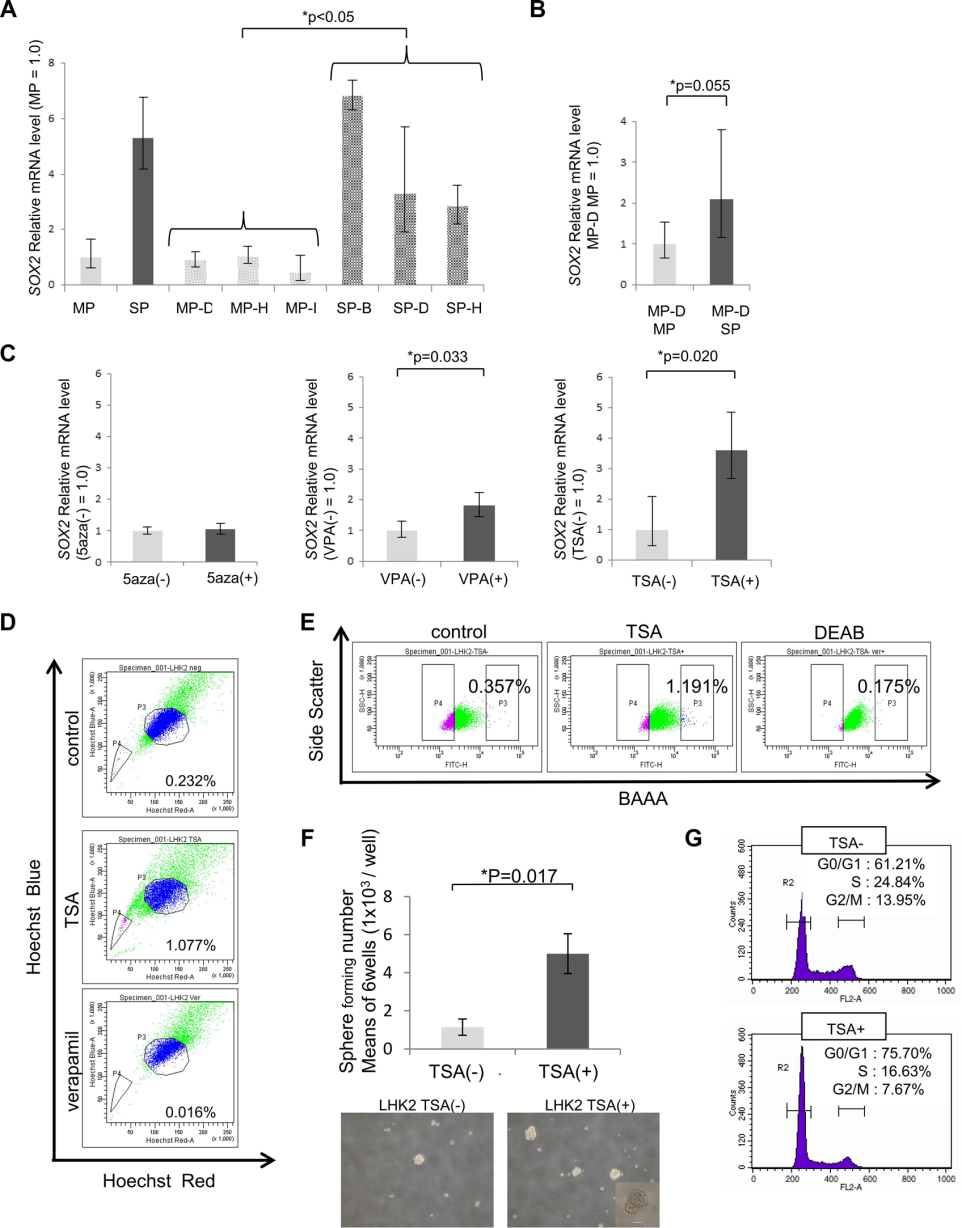
*SOX2* expression and stemness are regulated by class I *HDAC* (**A**) Quantitative real-time RT-PCR analysis of *SOX2* mRNA expression in LHK2 MP and SP cells, MP clone and SP clone cells, and MP and SP cells derived from MP-D clone cells. MP cells were used for the control, which was set as 1.0. Data are expressed as means ± s.d. of relative values compared with MP cells. Asterisks indicate significant differences. *P < 0.05. Steel-Dwass test. (**B**) Quantitative real-time PCR analysis of *SOX2* mRNA expression in LHK2 MP and SP cells derived from MP-D clone cells. MP cells derived from MP-D cells were used for the control, which was set as 1.0. Data are expressed as means ± s.d. of relative values compared with MP cells. Asterisks indicate significant differences. *P values. Student's *t*-test. (**C**) Quantitative real-time PCR analysis of *SOX2* mRNA expression in LHK2 cells treated with 5aza, VPA and TSA. Nontreated cells were used for the control, which was set as 1.0. Data are expressed as means ± s.d. of relative values compared with nontreated cells. Asterisks indicated significant differences. *P values. Paired *t*-test. (**D**) SP assay of LHK2 cells treated with TSA. The percentage represents the ratio of SP cells. (**E**) ALDEFLUOR assay of LHK2 cells treated with TSA. The percentage represents the ratio of ALDH^high^ cells. (**F**) Comparison of the numbers sphere-forming cells not treated with TSA (TSA(–)) and TSA-treated cells (TSA(+)) in LHK2 cells. Asterisks indicated significant differences. *P values. Paired *t*-test. Images of tumor spheres seeded with TSA(–) and TSA(+) in LHK2 cells. Scale bar, 100 μm. (**G**) Cell cycle analysis of cells not treated with TSA (TSA–) and TSA-treated cells (TSA+) in LHK2 cells.

Previously, we showed that *SOX2* was expressed in LHK2 SP cells at a higher level than that in LHK2 MP cells and that *SOX2* was involved in the maintenance of lung CSCs/CICs [18]. We thus investigated *SOX2* expression levels in LHK2 SP clone cells and MP clone cells by qRT-PCR. SP clone cells showed a significantly higher expression level of *SOX2* than that in MP clone cells, and MP clone cells showed low *SOX2* expression levels as in MP cells (Figure 2A). MP cells and SP cells derived from MP-D cells were also analyzed, and SP cells derived from MP-D cells showed a higher *SOX2* expression level than that in MP cells derived from MP-D cells, but the difference was not statistically significant (*p* = 0.055) (Figure 2B). These results indicate that a relatively high expression level of *SOX2* in the population might be important for production of an SP subpopulation.

Since all of the MP clones of LHK2 showed ability for dedifferentiation into SP cells in just one month of culture, we hypothesized that regulation of dedifferentiation might be controlled by epigenetic regulation, not by genetic events. To investigate the possible epigenetic regulation, we treated LHK2 cells with the DNA methyltransferase inhibitor 5′-aza-2′-deoxycytidine (5aza) and the histone deacetylase (*HDAC*) inhibitors valproic acid (VPA) and Trichostatin A (TSA) and examined the expression of *SOX2* by qRT-PCR. Treatment with 5aza did not change *SOX2* expression (Figure 2C). On the other hand, treatments with the *HDAC* inhibitors VPA and TSA resulted in significant enhancement of *SOX2* expression (Figure 2C). Since VPA is a class I *HDAC* inhibitor and TSA is a class I and class II *HDAC* inhibitor, *SOX2* expression might be controlled by class I *HDAC*.

To determine whether CSCs/CICs can be induced by an *HDAC* inhibitor, LHK2 cells were treated with TSA and examined by SP analysis and the ALDEFLUOR assay [22]. The ratio of SP cells was increased by TSA treatment (Figure 2D). Furthermore, aldehyde dehydrogenase 1 (*ALDH1*) high populations were increased by TSA treatment (Figure 2E). These observations indicate that CSCs/CICs might be induced by TSA treatment. To generalize these phenomena, other lung cancer cell lines of different histological subtypes were analyzed. Lung adenocarcinoma cell line A549, squamous cell carcinoma cell line Sq-1, large cell carcinoma cell line Lu99 and small cell carcinoma cell line Lc817 were treated with TSA, and *SOX2* expression and SP cells were investigated. A549 and Sq-1 cells showed significant enhancement of *SOX2* expression, and other cell lines also showed a tendency for *SOX2* expression enhancement (Supplementary Figure S3A). Furthermore, the ratios of SP cells were increased in all four cell lines by TSA treatment (Supplementary Figure S3B).

Since CSCs/CICs have sphere-forming ability in a floating culture condition [23], we performed a sphere forming assay using LHK2 and Sq-1 cells treated with TSA. Both LHK2 cells and Sq-1 cells showed stronger sphere formation ability in a TSA (+) condition than in a control condition (Figure 2F and Supplementary Figure S3C). Since CSCs/CICs show the quiescent stage in the cell cycle [8], we performed cell cycle analysis using LHK2 cells treated with TSA. TSA-treated LHK2 cells showed a tendency for quiescent stage maintenance (Figure 2G).

### The transcription factor *HOXA5* induces *SOX2* expression in the presence of a class I *HDAC* inhibitor

**Figure 3:**
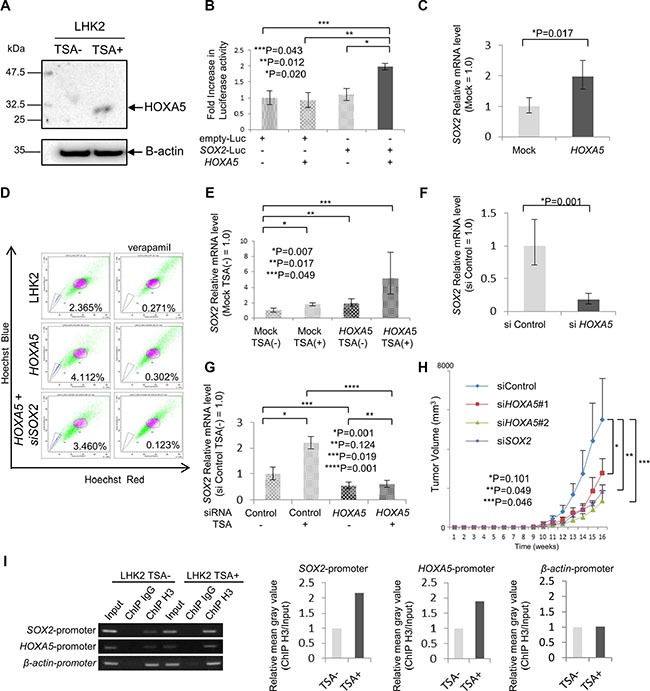
*HOXA5* has a role in *SOX2* expression and tumorigenicity (**A**) Expression of HOXA5 protein in LHK2 cells not treated with TSA (TSA–) and TSA-treated cells (TSA+) confirmed by Western blotting with an anti-HOXA5 antibody. (**B**) Luciferase assays for extracts of LHK2 cells transfected with *HOXA5* expression plasmid plus *SOX2* promoter–Luc constructs. The fold increase in Luciferase activity compared with cells transfected with an empty plasmid or empty-Luc plasmid was determined. Data are expressed as means ± s.d. Asterisks indicated significant differences. *P, **P values. Student's *t*-test. ***P values. Welch's *t*-test. (**C**) Quantitative real-time RT-PCR analysis of *SOX2* mRNA expression in LHK2 cells transfected with *HOXA5*. Asterisks indicated significant differences. *P values. Paired *t*-test. (**D**) SP assay of LHK2 cells transfected with *HOXA5* and si*SOX2*. The percentage represents the ratio of SP cells. (**E**) Quantitative real-time PCR analysis of *SOX2* mRNA expression in empty vector-transfected TSA(–) cells, empty vector-transfected TSA(+) cells, *HOXA5*-transfected TSA(–) cells and *HOXA5*-transfected TSA(+) cells. Asterisks indicated significant differences. *P, **P, ***P values. Paired *t*-test. (**F**) Quantitative real-time PCR analysis of *SOX2* mRNA expression in *HOXA5* siRNA-transfected LHK2 cells. Asterisks indicated significant differences. *P values. Paired *t*-test. (**G**) Quantitative real-time PCR analysis of *SOX2* mRNA expression in si Control-TSA(–) cells, si Control-TSA(+) cells, si *HOXA5*-TSA(–) cells and si *HOXA5*-TSA(+) cells. Asterisks indicated significant differences. *P, **P, ***P values. Paired *t*-test. (**H**) Tumor growth curves of siHOXA5 and siSOX2 transfected cells. derived from LHK2 cells injected in NOD/SCID mice, and representative views of mouse tumors. Each value is the mean tumor volume ± SD. *P, **P, ***P values. Paired *t*-test. (**I**) Binding of *SOX2* promoter and *HOXA5* promoter to acetylated histone shown by ChIP assay. β-actin was used as a positive control. Input: total Input DNA, ChIP IgG: normal rabbit IgG antibody, ChIP H3: acetyl-Histone H3 antibody. Right bar graph represents the relative mean grayvalue measured by ImageJ software (ChIP H3/Input).

*SOX2* expression was shown to be regulated by class I *HDAC*. To further investigate the gene expression mechanisms of *SOX2*, we searched for putative transcription factor-binding sites in the *SOX2* gene promoter region using SABiosciences' Text Mining Application and UCSC Genome Browser, and we found several candidate transcription factors (Supplementary Figure S4A). We investigated the expression of candidate transcription factors (*p300*, *SOX9*, *SOX5*, *POU3F2*, *FOXL1*, *HOXA5*, *zic2* and *Nanog*) in LHK2 SP cells and MP cells. *SOX5*, *POU3F2*, *HOXA5* and *Nanog* showed preferential expression in SP cells (Supplementary Figure S4B). Since the expression of *SOX2* was upregulated by class I *HDAC* inhibitors, regulation of the expression of candidate transcription factors by class I *HDAC* inhibitors was investigated. *SOX5*, *POU3F2*, *HOXA5* and *zic2* showed enhancement of expression in LHK2 cells treated with class I *HDAC* inhibitors (Supplementary Figure S4C). To generalize the gene expression in lung cancer cells, A549, Sq-1, Lu99 and Lc817 cells were treated with TSA, and the expression of transcription factors was investigated. Only *HOXA5* showed general expression (Supplementary Figure S4D and S4E). Protein expression of HOXA5 was detected in TSA-treated lung cancer cells (Figure 3A and Supplementary Figure S4F). These results indicate that *HOXA5* is a possible transcription factor for *SOX2* expression, and we thus further analyzed *HOXA5*. *HOXA5* has a single DNA-binding domain, and this gene was barely expressed in some normal adult tissues, lung cancer cell lines and primary lung cancer cells generally (Supplementary Figure S4G).

To investigate whether *HOXA5* induces the expression of *SOX2*, we performed a luciferase assay using luciferase DNA fused to the *SOX2* promoter region. Co-transfection with *HOXA5* induced significant luciferase activity compared to that in control cells (Figure 3B). To confirm the transcription activity of *HOXA5*, we transfected *HOXA5* cDNA into LHK2 cells and investigated *SOX2* expression by qRT-PCR. *HOXA5* transfection was confirmed by qRT-PCR (Supplementary Figure S4H) and we found that *HOXA5* transfection enhanced the expression of *SOX2* (Figure 3C). The ratio of SP cells was increased by *HOXA5* transfection (Figure 3D). Furthermore, we investigated whether treatment with TSA has an additional effect on *SOX2* expression in *HOXA5*-transfected LHK2 cells. *HOXA5* expression levels in empty vector-transfected TSA(–) cells, empty vector-transfected TSA(+) cells, *HOXA5*-transfected TSA(–) cells and *HOXA5*-transfected TSA(+) cells were confirmed by qRT-PCR (Supplementary Figure S4I). TSA treatment enhanced *SOX2* expression in both *HOXA5* non-transfected and transfected LHK2 cells, and *HOXA5*-transfected TSA(+) cells showed the highest *SOX2* expression (Figure 3E). SP cells increased by *HOXA5* transfection was cancelled by SOX2 knockdown using *SOX2* siRNA (Figure 3D).

To confirm the *SOX2* inducing role of *HOXA5*, a gene knockdown study using *HOXA5*-specific siRNA was performed. We designed *HOXA5*-specific siRNA and confirmed gene knockdown by qRT-PCR using *HOXA5* siRNA-transfected LHK2 cells (Supplementary Figure S4J). *SOX2* expression level was repressed by *HOXA5* gene knockdown (Figure 3F). Furthermore, we investigated whether knockdown of *HOXA5* repressed the induction of *SOX2* by TSA treatment. The expression of *HOXA5* in control siRNA-transfected TSA(–) cells, control siRNA-transfected TSA(+) cells, *HOXA5* siRNA-transfected TSA(–) cells and *HOXA5* siRNA-transfected TSA(+) cells was examined by qRT-PCR (Supplementary Figure S4K). And we found that *HOXA5* gene knockdown also cancelled the *SOX2* expression induced by TSA treatment (Figure 3G). *HOXA5* knockdown by siRNA suppressed the sphere-forming ability of LHK2 cells (Supplementary Figure S4L). Furthermore, *HOXA5* knockdown by siRNA suppressed the tumorigenicity of LHK2 cells as well as *SOX2* knockdown (Figure 3H). To investigate whether *SOX2* and *HOXA5* promoter regions bind to acetylated histone, we performed a ChIP-PCR assay using an acetyl-Histone H3 antibody. Consistent with the results of qRT-PCR, the DNA fragments including each of the *SOX2* and *HOXA5* promoter regions encompassing the acetylated histone were pulled down more in the cells treated with TSA (Figure 3I).

### *HOXA5* represses expression of the tumor suppressor gene *TP53* in lung cancer

**Figure 4:**
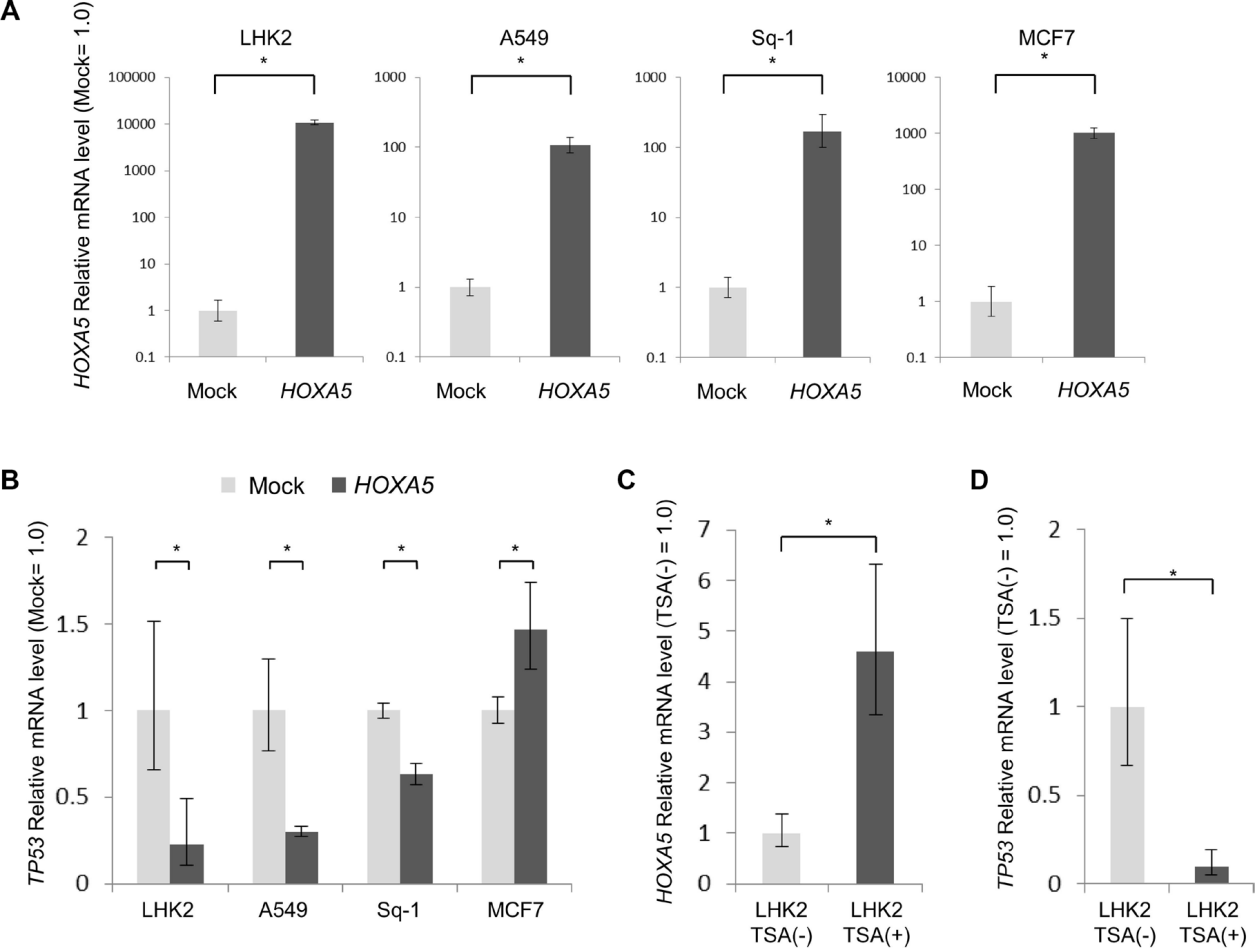
*HOXA5* repress *TP53* expression in lung cancer (**A**) Quantitative real-time PCR analysis of *HOXA5* mRNA expression in LHK2, A549, Sq-1 and MCF7 cells transfected with *HOXA5*. Empty vector-transfected cells were used for the control, which was set as 1.0. Data are expressed as means ± s.d. of relative values compared with empty vector-transfected cells. Asterisks indicated significant differences. *P < 0.05. *t*-test. (**B**) Quantitative real-time PCR analysis of *TP53* mRNA expression in *HOXA5*-transfected cells (LHK2, A549, Sq-1 and MCF7 cells). Asterisks indicated significant differences. *P < 0.05. *t*-test. (**C**) (**D**) Quantitative real-time PCR analysis of *HOXA5* and *TP53* mRNA expression in LHK2 treated with TSA. Cells not treated with TSA were used for the control, which was set as 1.0. Data are expressed as means ± s.d. of relative values compared with nontreated cells. Asterisks indicated significant differences. *P < 0.05. *t*-test.

It was reported that the tumor suppressor gene *TP53* is activated by *HDAC* inhibitors [24–26], and if the status of *TP53* is wild type, it will have a suppressive effect on tumor progression [27–30]. The status of *TP53* in LHK2 cells was in fact analyzed by a next-generation DNA sequencer, and it was found that LHK2 cells have wild-type *TP53* (data not shown). In addition, it was reported that *HOXA5* promotes *TP53* expression in breast cancer and other cancers [31–33], and *TP53* was reported to suppress cancer stemness [34]. We thus investigated whether *HOXA5* promotes *TP53* expression in lung cancer cells. We transfected *HOXA5* cDNA in lung cancer cell lines (LHK2, A549 and Sq-1 cells) and breast cancer cell lines (MCF7 cells) (Figure 4A). It was found that A549 and Sq-1 cells also have wild-type *TP53* (data not shown). We analyzed *TP53* expression levels by qRT-PCR. The expression of *TP53* was significantly repressed by *HOXA5* cDNA transfection in lung cancer cell line, whereas the expression of *TP53* was significantly enhanced by *HOXA5* cDNA transfection in breast cancer cell line as reported previously (Figure 4B) [31]. Furthermore, treatment of LHK2 cells with TSA enhanced the expression of *HOXA5* (Figures 3B and 4C) and repressed the expression of *TP53* (Figure 4D).

A previous study showed that the *HOXA5* promoter region is methylated in breast cancer cells and that lack of *HOXA5* expression may be one mechanism underlying the loss of *TP53* expression [31]. In fact, MCF7 cells treated with the DNA methyltransferase inhibitor 5aza showed increased a significantly expression level of *HOXA5* (Supplementary Figure S5). On the other hand, treatment with 5aza did not increase the expression level of *TP53* in lung cancer cell lines (Supplementary Figure S5). The difference of epigenetic regulation in the *HOXA5* promoter region may control the expression of *TP53*.

### Repression of *HDAC8* by oxidative stress is related to acquisition of stemness in lung cancer

We showed that lung cancer cells dedifferentiate into CSCs/CICs by epigenetics, and we also investigated the factor inducing the dedifferentiation. Lung cancer frequently develops in patients with chronic obstructive pulmonary disease (COPD), an inflammatory lung disease, and it has recently been reported that etiology of COPD is related to the expression of inflammatory genes induced by histone acetylation due to oxidative stress [35, 36]. We examined whether LHK2 cells acquire stemness by oxidative stress and increase the expression of *HOXA5* and *SOX2*.

**Figure 5:**
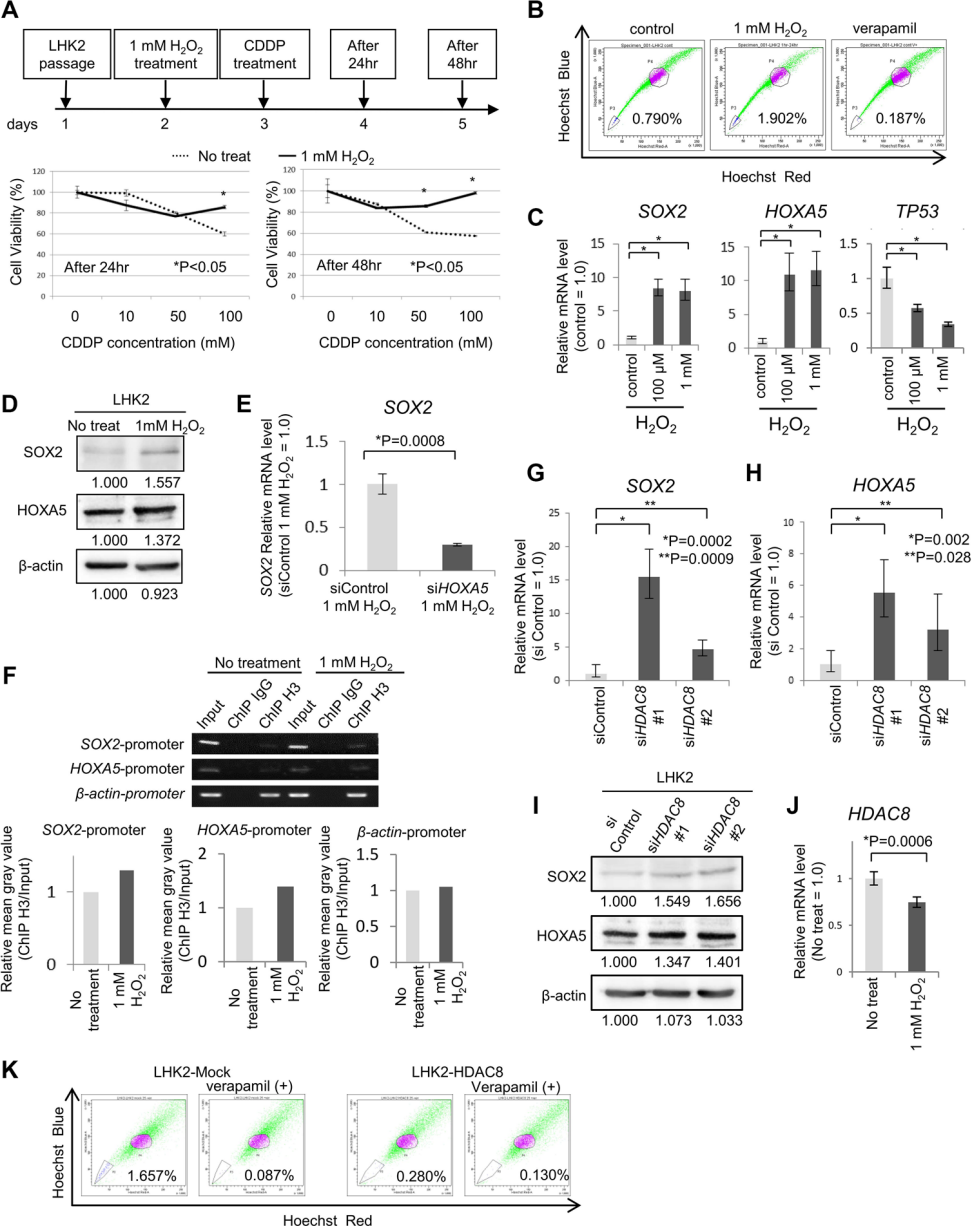
Repression of *HDAC8* by oxidative stress may be related to acquisition of stemness (**A**) Cell proliferation assays were performed in LHK2 cells treated with 1 mM H_2_O_2_ for 1 hr and cisplatin (CDDP) by using a Cell Counting Kit-8 and analyzed after 24 hr and 48 hr. (**B**) LHK2 cells were treated with 1 mM H_2_O_2_ for 1 hr and analyzed by SP assay after 24 hr. The percentage represents the ratio of SP cells. (**C**) Quantitative real-time PCR analysis of *HOXA5*, *SOX2* and *TP53* mRNA expression in LHK2 cells treated with 100 μM for 1 hr and 1 mM H_2_O_2_ for 1 hr. Non-treated cells were used for the control, which was set as 1.0. Data are expressed as means ± s.d. of relative values compared with nontreated cells. Asterisks indicated significant differences. *P < 0.05. *t*-test. (**D**) Expression of SOX2 and HOXA5 protein in LHK2 cells not treated with H_2_O_2_ (No treat) and 1 mM H_2_O_2_-treated cells (1 mM H_2_O_2_) confirmed by Western blotting with an anti-SOX2 antibody and an anti-HOXA5 antibody. **(E**) Quantitative real-time RT-PCR analysis of *SOX2* mRNA expression in *HOXA5* siRNA-transfected cells treated with 1 mM H_2_O_2_ for 1 hr. Control siRNA-transfected cells treated with 1 mM H_2_O_2_ cells were used for the control, which was set as 1.0. Data are expressed as means ± s.d. of relative values compared with control cells. Asterisks indicated significant differences. *P values. *t*-test. (**F**) Binding of *SOX2* promoter and *HOXA5* promoter to acetylated histone shown by ChIP assay. β-actin was used as a positive control. Input: total Input DNA, ChIP IgG: normal rabbit IgG antibody, ChIP H3: acetyl-Histone H3 antibody. Right bar graph represents the relative mean gray value measured by ImageJ software (ChIP H3/Input). (**G**) Quantitative real-time PCR analysis of *SOX2* mRNA expression in *HDAC8* siRNA-transfected LHK2 cells. Asterisks indicated significant differences. *P, **P values. *t*-test. (**H**) Quantitative real-time PCR analysis of *HOXA5* mRNA expression in *HDAC8* siRNA-transfected LHK2 cells. Asterisks indicated significant differences. *P, **P values. *t*-test. (**I**) Expression of SOX2 and HOXA5 protein in *HDAC8* siRNA-transfected LHK2 cells confirmed by Western blotting with an anti-SOX2 antibody and an anti-HOXA5 antibody. (**J**) Quantitative real-time PCR analysis of *HDAC8* mRNA expression in LHK2 cells treated with 1 mM H_2_O_2_ for 1 hr. Asterisks indicated significant differences. *P values. *t*-test. (**K**) SP analysis of *HDAC8* overexpressed LHK2 cells. The percentage represents the ratio of SP cells.

We confirmed oxidative stress by enhancement of the level of reactive oxygen species (ROS) using treatment with hydrogen peroxide (H_2_O_2_) for one hour in LHK2 cells. Each concentration of H_2_O_2_ increased the level of ROS (Supplementary Figure S6A). Many of the cells died with 10 mM H_2_O_2_ treatment, and following experiments were performed using 100 μM and 1 mM H_2_O_2_. First we confirmed that cells subjected to oxidative stress acquired chemoresistance ability as stemness. The results showed that LHK2 cells treated with 1 mM H_2_O_2_ had higher cell viability rates than those of control cells (Figure 5A). Furthermore, treatment with H_2_O_2_ increased the ratio of SP cells compared with that in control cells (Figure 5B).

We then examined *SOX2* and *HOXA5* expression levels in LHK2 cells treated with H_2_O_2_. Each concentration of H_2_O_2_ increased the expression of SOX2 and HOXA5 (Figure 5C and 5D). The protein expressions of SOX2 and HOXA5 is smaller than those in transcripts. This might depend on the efficacy of translation and the stability of proteins. *TP53* expression was decreased by H_2_O_2_ (Figure 5C). Furthermore, we investigated whether knockdown of *HOXA5* repressed the induction of *SOX2* by oxidative stress. We examined the *HOXA5* expression level in *HOXA5* knockdown cells treated with 1 mM H_2_O_2_ by qRT-PCR (Supplementary Figure S6B), and we found that knockdown of *HOXA5* cancelled the induction of *SOX2* expression by treatment with 1 mM H_2_O_2_ (Figure 5E).

To determine whether *SOX2* and *HOXA5* promoter regions bind to acetylated histone in cells subjected to oxidative stress, we performed a ChIP-PCR assay using an acetyl-Histone H3 antibody. The results showed that DNA fragments including each of the *SOX2* and *HOXA5* promoter regions encompassing the acetylated histone were pulled down more in cells treated with 1 mM H_2_O_2_ (Figure 5F).

We then investigated which type of class I *HDAC* is related to the expression of *SOX2*. Class I *HDAC*s consist of *HDAC1*, *2*, *3* and *8*, and we performed gene knockdown using *HDAC*-specific siRNAs. We designed two different specific siRNAs in *HDAC1*, *HDAC2*, *HDAC3* and *HDAC8* and confirmed gene knockdown by qRT-PCR using LHK2 cells transfected with each of the *HDAC* siRNAs (Supplementary Figure S6C). Knockdown of *HDAC1* and *HDAC2* did not change the expression of *SOX2* (Supplementary Figure S6D). One of the *HDAC3*-specific siRNAs increased the expression of *SOX2*, but the other did not (Supplementary Figure S6D). LHK2 cells transfected with two different *HDAC8-*specific siRNAs showed higher expression levels of SOX2 than those in control siRNA-transfected cells (Figure 5G and 5I). On the other hand, *HDAC8* overexpression decreased the SP cells in LHK2 cells (Figure 5K). HOXA5 expression was also increased by *HDAC8* knockdown (Figure 5H and 5I). *HDAC8* siRNA#1 and siRNA#2 decreased *HDAC8* at similar levels; however, the expression levels of SOX2 and HOXA5 were different. This might be off-target effect of siRNAs. Finally, we examined the expression of class I *HDACs* by treatment with oxidative stress. Only *HDAC8* was significantly repressed by treatment with 1 mM H_2_O_2_, suggesting that repression of *HDAC8* by oxidative stress is the initial event for acquiring lung cancer stemness (Figure 5J and Supplementary Figure S6E).

## DISCUSSION

Cancer is composed of heterogeneous subpopulations with regard to morphology and function. The ‘cancer stem cell model’ and ‘clonal evolution model’ were two major models to explain the heterogeneity of cancer [8, 37–39]. Recent studies have revealed that some differentiated non-CSCs/CICs can re-obtain a CSCs/CICs phenotype by various stimulations including microenvironment, and a ‘dynamic CSC model’ has been proposed to explain the plasticity of non-CSCs/CICs [40]. In this study, we analyzed the differentiation status and dedifferentiation status of human lung cancer cells at the single cell level and showed that some lung differentiated MP cells dedifferentiate into CSCs/CICs-like SP cells in short-term culture with relative high expression level of *SOX2*. Our data suggest that human lung cancer cells have plasticity by which lung differentiated non-CSCs/CICs can dedifferentiate into CSCs/CICs *in vitro*. Thus, the ‘dynamic CSC model’ might be a valid model for lung cancers.

Chaffer et al. showed that human mammary normal and neoplastic non-stem cells can convert to stem-like cells, suggesting plasticity of mammary epithelial cells [14]. Previous studies have shown that microenvironments including transforming growth factor-β (TGF-β) and hepatocyte growth factor (HGF) have roles in the plasticity of breast cancer cells and colon cancer cells, respectively [17, 41]. In our study, we showed that oxidative stress might be a mechanism by which dedifferentiation of non-CSCs/CICs is induced. Cigarette smoking is one of the risk factors of lung cancer [42], and cigarette smoke includes oxidants with other chemical carcinogens. Cigarette smoke thus might be one source for oxidative stress for carcinogenesis in smokers. Inflammation induced by respiratory diseases including COPD and Interstitial pneumonia is another risk factor of lung cancer [43, 44]. Immune cells including neutrophils and macrophages produce reactive oxygen species (ROS). Thus, ROS produced by immune cells might be another source of oxidative stress.

In a previous study, we showed that *SOX2* is expressed in lung CSCs/CICs and that *SOX2* has an essential role in the maintenance of lung CSCs/CICs [18]. We showed that the expression of *SOX2* is upregulated more in dedifferentiated CSCs/CICs derived from non-CSCs/CICs than in non-CSCs/CICs, indicating that *SOX2* might be a responsible key molecule in the dedifferentiation of lung cancer cells. Activation of epithelial-mesenchymal transition (EMT) and Wnt/β-Catenin signaling have been shown to have roles in dedifferentiation of breast cancer and colon cancer, respectively [16, 17, 41]. Thus, different molecular mechanisms are involved in dedifferentiation in different types of cancers.

*SOX2* and its partner *POU5F1* (*OCT3/4*) complex has transcriptional activity of *SOX2* itself in ES cells [45]. However, the transcription factors of *SOX2* in cancer cells have remained unknown. In this study, we investigated the gene expression mechanisms of *SOX2* and found by a luciferase assay that *HOXA5* is one of the transcription factors to induce *SOX2* expression. Treatment by TSA increased the expression of *SOX2* under overexpression of *HOXA5*. Thus another transcription factor derived by TSA or histone acetylation status of *SOX2* promoter may also involved in the expression of *SOX2*. However, *HOXA5* was reported to be a tumor-suppressor gene that can induce a *TP53* tumor-suppressor gene [31] and that it is related to lung development [46]. Previous studies showed that the promoter region of *HOXA5* is inactivated by DNA methylation in breast and lung cancer cells [31, 47]. However, our results showed that *HOAX5* transcription in lung cancer cells was not induced by 5aza treatment, whereas *HOAX5* transcription was induced by 5aza treatment in breast cancer cells. These observations indicate that the methylation status of the *HOAX5* promoter region is variable and it might depend on several conditions. We found that the *HOXA5* promoter was regulated by acetylation of H3 that can be induced by repression of *HDAC8* expression by oxidative stress. Thus, oxidative stress might play a role in induction of lung CSCs/CICs by histone acetylation and inducing *HOXA5* expression followed by *SOX2* expression and *TP53* repression. Lungs are always exposed to oxidative stress, and oxidative stress was shown to induce histone acetylation by repression of *HDAC2* in the lung adenocarcinoma cell line A549 [35, 36]. These observations indicate that oxidative stress can modulate epigenetic gene expression regulation by repressing *HDAC*s; however, the molecular mechanisms are still elusive.

Recently, *HDAC* inhibitors have been expected to have effects as adjuvants for tumors [48–50] and it is thought that *TP53* is related to the effect [51]. Since it had been reported that *TP53* is induced by *HOXA5* [31], we investigated the interaction between them. Interestedly, *HOXA5* induced wild-type *TP53* in breast cancer cells as described previously. On the other hand, *HOXA5* repressed wild-type *TP53* expression in lung cancer cells. The molecular mechanisms by which *HOXA5* showed different functions are still unknown; however, repression of *TP53* might be one of the mechanisms by which CSCs/CICs are induced by *HOXA5* in lung cancer cells. *SOX2* is expressed in some normal adult tissues including brain [18], however, *HOXA5* is only upregulated under the induction of *SOX2* expression. These observations suggest that *HOXA5* can be a reasonable target for CSC/CIC-targeting therapy.

**Figure 6:**
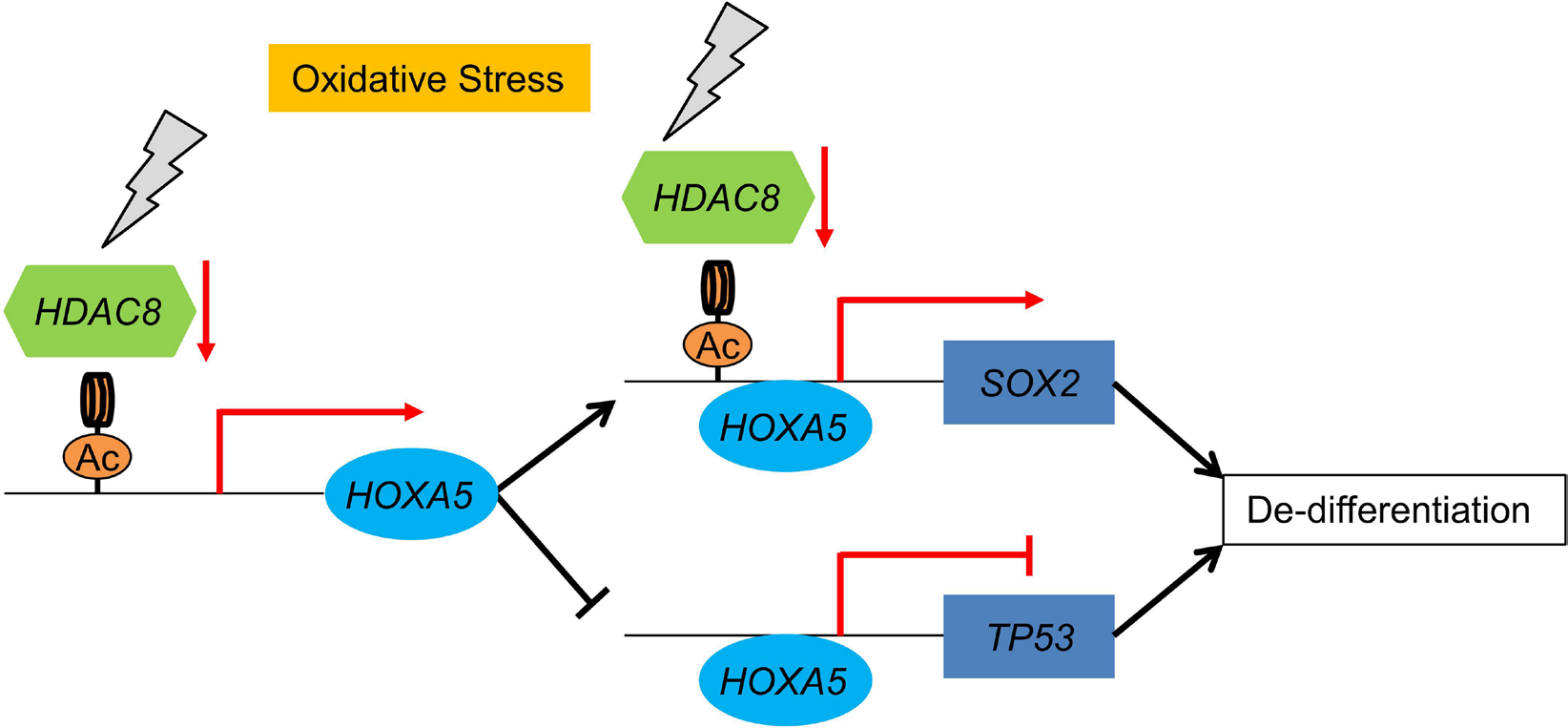
Schematic summary of lung cancer cell dedifferentiation model Oxidative stress represses the expression of *HDAC8*, resulting in an increase in the acetylation of histone H3 of *HOXA5* and *SOX2* promoter regions. Upregulated *HOXA5* induces the transcription of *SOX2* and represses the transcription of *TP53*.

Taken together, lung non-CSCs/CICs can dedifferentiate into lung CSCs/CICs by histone acetylation. Histone acetylation induces the expression of *HOXA5*, resulting in repression of *TP53* expression and induction of *SOX2* expression, which is responsible for the maintenance of CSCs/CICs. Histone acetylation can be induced by repression of *HDAC8* expression by oxidative stress. These observations suggest that lung cancer cells can be dedifferentiated by oxidative stress and that the transcription factor *HOXA5* has a critical role and *HOXA5* can be a reasonable molecular target of lung CSC/CIC-targeting therapy (Figure 6).

## MATERIALS AND METHODS

### Ethics statement

Mice were maintained and experimented on in accordance with the guidelines after approval by the Committee of Sapporo Medical University (No.10-032).

### Side population (SP) assay

Side population (SP) cells were isolated as described previously using Hoechst 33342 dye (Lonza, Basel, Switzerland) with some modifications [19, 20]. Briefly, cells were resuspended at 1 × 10^6^/mL in pre-warmed DMEM supplemented with 5% FBS. Hoechst 33342 dye was added at a final concentration of 2.5 μg/mL in the presence or absence of verapamil (75 μM; Sigma-Aldrich) and the cells were incubated at 37°C for 60 min or 90 min with intermittent shaking. Analyses and sorting were performed with a FACSAria II cell sorter (Becton Dickinson). The Hoechst33342 dye was excited at 357 nm and its fluorescence was analyzed using dual wave-lengths (blue, 402–446 nm; red, 650–670 nm).

### Method for establishing SP clone cells and MP clone cells

SP cells and MP cells were isolated from LHK2 cells as described above and were plated at a single cell per well in a 96-well plate. Sorted single cells were cultured in DMEM supplemented with 10% FBS at 37°C in a humidified 5% CO_2_ atmosphere, and SP clone cells and MP clone cells were obtained after one week. We also established SP clone cells and MP clone cells derived from one population of MP clone cells by the same method.

### Quantitative real-time PCR analysis

Quantitative real-time PCR was performed using a StepOne and StepOnePlus Real-Time PCR System (Applied Biosystems, Foster City, CA) according to the manufacturer's protocol. Primers and probes were designed by the manufacturer (TaqMan gene expression assays; Applied Biosystems). Thermal cycling was performed using 40 or 45 cycles of 95°C for 15 sec followed by 60°C for 1 min. Each experiment was done in triplicate, with normalization to the *GAPDH* gene as an internal control.

### Methods for treatment with 5′–aza-2′-deoxycytidine, valproic acid and Trichostatin A

Cells were treated with 5′-aza-2′-deoxycytidine (5aza) (5 μM; Sigma-Aldrich), valproic acid (VPA) (4 mM; Sigma-Aldrich) and Trichostatin A (TSA) (100 nM; Sigma-Aldrich) according to the manufacturer's protocol.

### Cell transfection

An expression vector of pcDNA3.1 harboring *HOXA5* cDNA was used for transfection into LHK2 cells. Transfection of cells was performed with Lipofectamine 2000 (Invitrogen, Carlsbad, CA, USA) following the manufacturer's protocol. For stable transfection of *HOXA5* and *HDAC8* genes, a retrovirus vector pMXs-puro was used as described previously [21].

### Luciferase assay

The *SOX2* promoter connected downstream to the Luciferase gene was purchased from SwitchGear Genomics, a pCMV-LacZ vector was purchased from Clontech, and an expression vector of pcDNA3.1 harboring *HOXA5* cDNA was established in our laboratory. Luciferase assay was performed using Luciferase Assay System (Promega) according to the manufacturer's protocol, and β-galactosidase assay as an internal control was performed using a High Sensitivity β-Galactosidase Assay Kit (Agilent Technologies) according to the manufacturer's protocol.

### Small interfering RNA transfection

*HOXA5* small interfering RNA (siRNA) was designed and synthesized using the BLOCK-it RNAi designer system (Life Technologies). The oligonucleotide encoding *HOXA5* siRNA was 5′- AUUGCUCGCUCACG GAACUAUGAUC −3′. Cells were seeded at 50% confluence, and transfections were carried out using Lipofectamine 2000 (Invitrogen, Carlsbad, CA, USA) in Opti-MEM according to the manufacturer's protocol.

### Chromatin immunoprecipitation (ChIP) assay

ChIP assays were performed using the Acetyl-Histone H3 Immunoprecipitation (ChIP) Assay Kit (Upstate) according to the manufacturer's protocol. In brief, 1 × 10^6^ TSA-treated and untreated cells, and bulk and 1 mM H_2_O_2_ for 1 hr treated cells were cross-linked by adding formaldehyde directly to the culture medium. Cells were harvested and sonicated to shear DNA to lengths between 100 and 200 bp. After centrifuging samples for 10 min at 13,000 rpm at 4°C, the supernatant was pre-cleared with 80 μl of Salmon Sperm DNA/Protein A-Agarose-50% Slurry for 30 min at 4°C with agitation. Two μl of Acetyl-Histone H3 antibody was then added to the supernatant fraction for incubation overnight at 4°C with rotation. Then 60 μl of Salmon Sperm DNA/Protein A-Agarose was added to collect the antibody-histone complex. The protein A-agarose-antibody-histone complex was extensively washed for 5 min as suggested and then eluted with elution buffer and heated at 65°C for 6 h to reverse histone-DNA crosslinks. The DNA was recovered by phenol/chloroform extraction and ethanol precipitation. PCR was performed using two pairs of primers (*SOX2* promoter: 5′- CAATGACACACCAACTCCTGC −3′ and 5′- CACACGCCTTTTCGAAGGAA −3′; *HOXA5* promoter: 5′- TCAAGGAGAACCCTCCGACT −3′ and 5′- TGTTTCTCCAAGGCGAGGTC −3′; β-Actin: 5′- CCAGAGCAAGAGAGGCATCC −3′ and 5′- AGAGTCCTACGGAAAACGGC −3′).

### Xenograft transplantation in NOD/SCID mice

LHK2 cells traesfected with control siRNA, *HOXA5* siRNA and *SOX2* siRNA were resuspended at concentrations of 1 × 10^3^ cells in phosphate buffered saline and Matrigel (BD Biosciences) mixture (1:1), and were injected subcutaneously into the right and left mid back areas of anesthetized non-obese diabetic/severe combined immunodeficient (NOD/SCID) female mice (Charles River Laboratory Japan, Yokohama, Japan) at the age of 4–6 weeks. Tumor growth was monitored weekly, and tumor volume was calculated by XY^2^ / 2 (X = long axis, Y = short axis).

### Cell proliferation assay

Cell proliferation assays of LHK2 cells were performed by using a Cell Counting Kit-8 (Dojindo, Kumamoto, Japan). Cells were plated in 96-well plates at 1 × 10^4^ cells per well and cultured in DMEM (SIGMA, Ishikari, Japan) supplemented with 10% FBS (Life Technologies Japan, Tokyo, Japan) at 37°C in a humidified 5% CO_2_ atmosphere. The next day, LHK2 cells were treated with 1 mM H_2_O_2_ or not treated, and on the third day, they were treated with cisplatin (CDDP) at different concentrations. The cell numbers in triplicate wells were measured as the absorbance (450 nm) of reduced WST-8 (2-(2-methoxy-4-nitrophenyl)-3-(4-nitrophenyl)-5-(2,4-disulfophenyl)-2H-tetrazolium, monosodium salt).

## SUPPLEMENTARY MATERIALS FIGURES


