# Supplementary material for: Plasticity of lung cancer stem-like cells is regulated by the transcription factor HOXA5 that is induced by oxidative stress
Source: Oncotarget. 2016 Jul 13;7(31):50043–56. doi: 10.18632/oncotarget.10571 (PMC5226567; doi:10.18632/oncotarget.10571)
Supplement: Supplementary file 1 [file oncotarget-07-50043-s001.pdf]

Plasticity of lung cancer stem-like cells is regulated by the transcription factor *HOXA5* that is induced by oxidative stress

Supplementary Materials

SUPPLEMENTARY MATERIALS AND METHODS

Cell lines and cell culture

The human lung adenocarcinoma cell line LHK2 was established in our laboratory [1]. The human lung adenocarcinoma cell line A549 was purchased from American Type Culture Collection (Manassas, VA, USA). The human lung squamous cell carcinoma cell line Sq-1 and human large cell carcinoma cell line Lu99 were obtained from the Cell Resource Center for Biomedical Research, Tohoku University (Sendai, Japan). The human lung small cell carcinoma cell line Lc817 was purchased from the Japanese Cancer Research Resources Bank. The human breast adenocarcinoma cell line MCF7 was purchased from American Type Culture Collection (Manassas, VA, USA). All of these cancer cells were cultured in DMEM (SIGMA, Ishikari, Japan) supplemented with 10% FBS (Life Technologies Japan, Tokyo, Japan) at 37°C in a humidified 5% CO_2 atmosphere.

Isolation of primary cancer cells from clinical specimens

All studies were approved by Institutional Review Boards (IRB) of Sapporo Medical University Hospital. Written Informed consent was obtained from all patients according to the guidelines of the Declaration of Helsinki. Cell lines were established from three primary lung cancers including two pleural effusions and one pericardial effusion. Pleural and pericardial effusions were collected, pelleted, washed and resuspended in Iscove's Modified Dulbecco's Medium (IMDM, Life Technologies). These cell lines were maintained in IMDM supplemented with 10% FBS (Life Technologies Japan, Tokyo, Japan) at 37°C in a humidified 5% CO_2 atmosphere.

ALDEFLUOR assay

Aldehyde dehydrogenase (ALDH) activity was detected using an ALDEFLUOR assay kit (StemCell Technologies) according to the manufacturer's protocol [2]. Cells were stained with bodipyaminoacetaldehyde (BAAA) at 1.5 mM and incubated for 30 min at 37°C. An inhibitor of ALDH1, diethylamino -benzaldehyde (DEAB), at a 10-fold molar excess was used as a negative control. One million stained cells were analyzed by FACS Aria II. High fluorescent ALDH1-expressing cells (ALDH1 high) were detected in the green fluorescence channel (520–540 nm).

Sphere formation assay

A spherical colony formation assay was performed using a stem cell medium (DMEM/F12 medium, Life Technologies) supplemented with 20 ng/mL human EGF (Life Technologies) and 20 ng/mL human basic fibroblast growth factor (Sigma). TSA-treated and non-treated LHK2 cells were plated at 1×10^3 cells per well in 6-well ultra-low attachment plates (Corning Inc., Corning, NY, 14831) and cultured for about 2–3 weeks. The morphology of the cells was assessed and pictures were taken under a light microscope. Round cell clusters larger than 100 μm were judged as spheres.

RT-PCR analysis

Reverse transcription - polymerase chain reaction (RT-PCR) analysis was performed as described previously [3]. Total RNA (tRNA) was isolated from cultured cells by an RNeasy Mini Kit (Qiagen, Valencia, CA, USA) using DNase (Qiagen) according to the manufacturer's protocol. cDNA was synthesized using Superscript III and oligo (dT) primer (Invitrogen) according to the manufacturer's protocol. PCR amplification was performed with PrimeSTAR HS DNA polymerase (Takara Biotechnology, Japan) for *SOX2* and with Taq DNA polymerase (QIAGEN, Duesseldorf, Germany) for others. The PCR mixture was initially incubated at 98°C for 2 min, followed by 35 cycles of denaturation at 98°C for 15 sec, and annealing and extension at 68°C for 30 sec for *SOX2*, and the other PCR mixtures were initially incubated at 94°C for 2 min, followed by 35 cycles of denaturation at 94°C for 15 sec, annealing at 58°C for 30 sec and extension at 72°C for 30 sec.

The PCR products were visualized with ethidium bromide staining under UV light after electrophoresis on 1.5% agarose gel. Primer pairs used for RT-PCR analysis were 5′-CATGATGGAGACGGAGCTGA-3′ and 5′-ACCCCGCTCGCCATGCTATT-3′ for *SOX2* with an expected PCR product size of 410 bp, 5′-ACCCACATCAGCAGCAGAG- 3′ and 5′-GAACTCC TTCTCCAGCTCCA-3′ for *HOXA5* with an expected PCR product size of 240 bp, and 5′-ACCACAGTCCATGC CATCAC-3′ and 5′-TCCACCACCCTGTTG CTGTA-3′ for glyceraldehyde-3-phosphate dehydrogenase (*GAPDH*) with an expected product size of 452 bp. *GAPDH* was used as an internal control.

Small interfering RNA transfection

HDAC small interfering RNA (siRNA) was designed and synthesized using the BLOCK-it RNAi designer system (Life Technologies). The oligonucleotide encoding *HDAC1* siRNA #1 was 5′- AAGTCCGAGGCA TCTGGCTTCTGTT -3′, *HDAC1* siRNA #2 was 5′- GATC GGTTAGGTTGCTTCAATCTAA -3′ , *HDAC2* siRNA #1 was 5′- CGCATGACCCATAACTTGCTGTTAA -3′, *HDAC2* siRNA #2 was 5′- GGCTGTTAATTGGGC TGGAGGATTA -3′, *HDAC3* siRNA #1 was 5′- CCAGAT CCGCCAGACAATCTTTGAA -3′, *HDAC3* siRNA #2 was 5′- GGGATGGCATTGATGACCAGAGTTA -3′, *HDAC8* siRNA #1 was 5′- TCGCTGGTCCCGGTTTATA TCTATA -3′, and *HDAC8* siRNA #2 was 5′- CGATGAT GATCATCCGGACTCCATA -3′ . Cells were seeded at 50% confluence, and transfections were carried out using Lipofectamine 2000 (Invitrogen, Carlsbad, CA, USA) in Opti-MEM according to the manufacturer’s protocol.

Cell cycle analysis

After the cells has been fixed with 70% ethanol, they were resuspended in PBS containing 250 mg/ml RNase A (Sigma-Aldrich) for 30 min at 37°C and stained with 50 mg/ml propidium iodide for 10 min at 4°C in the dark. Stained cells were analyzed with a FACSCalibur (Becton Dickinson), and the data were analyzed using the Mod-Fit cell cycle analysis program.

Western blotting

Cell lysate with SDS sample buffer was separated by denaturing SDS-PAGE. Separated proteins were transferred onto nitrocellulose membranes and probed with rabbit anti-HOXA5 polyclonal antibody (ASB) and mouse anti-SOX2 monoclonal antibody (ABG). β-Actin was used as a loading control and was detected with a rabbit pAb (Sigma). Anti-HOXA5 antibody was used at 2000-times dilution and Anti-SOX2 antibody was used at 200-times dilution and anti-β-actin antibody was used at 5000-times dilution.

Statistical analysis

Statistical analysis between two groups with multiple populations of real-time PCR was performed by the multiple comparison test for non-parametric data (Steel-Dwass test). Statistical analyses between two groups of real-time PCR, sphere formation assay and Luciferase Assay were performed by Student’s *t*-test, Welch’s *t*-test and paired *t*-test.

REFERENCES

1. Hirohashi Y, Torigoe T, Hirai I, Tamura Y, Nakatsugawa M, Inoue Y, Kanaseki T, Kamiguchi K, Ikeda H, Sasaki A, Yamanaka N, Sato N. Establishment of shared antigen reactive cytotoxic T lymphocyte using co-stimulatory molecule introduced autologous cancer cells. Exp Mol Pathol. 2010; 88:128–132.
2. Ginestier C, Hur MH, Charafe-Jauffret E, Monville F, Dutcher J, Brown M, Jacquemier J, Viens P, Kleer CG, Liu S, Schott A, Hayes D, Birnbaum D, et al. ALDH1 is a marker of normal and malignant human mammary stem cells and a predictor of poor clinical outcome. Cell stem cell. 2007; 1:555–567.
3. Nakatsugawa M, Hirohashi Y, Torigoe T, Asanuma H, Takahashi A, Inoda S, Kiriyama K, Nakazawa E, Harada K, Takasu H, Tamura Y, Kamiguchi K, Shijubo N, et al. Novel spliced form of a lens protein as a novel lung cancer antigen, Lengsin splicing variant 4. Cancer science. 2009; 100:1485–1493.

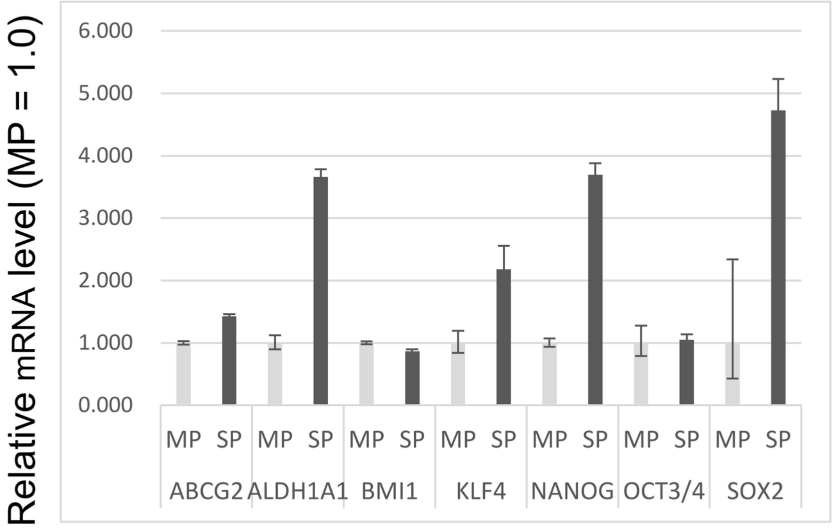

Supplementary Figure S1: Gene expression profiles in SP cells and MP cells derived from LHK2 cells.Quantitative real-time PCR analysis of *ABCG2, ALDH1A1, BMI1, KLF4, NANOG, OCT3/4* and *S0X2* mRNAs expression in SP cells and MP cells derived from LHK2 cells were performed. The expressions in MP cells were used for the control, which was set as 1.0. Data are expressed as means ± s.d.

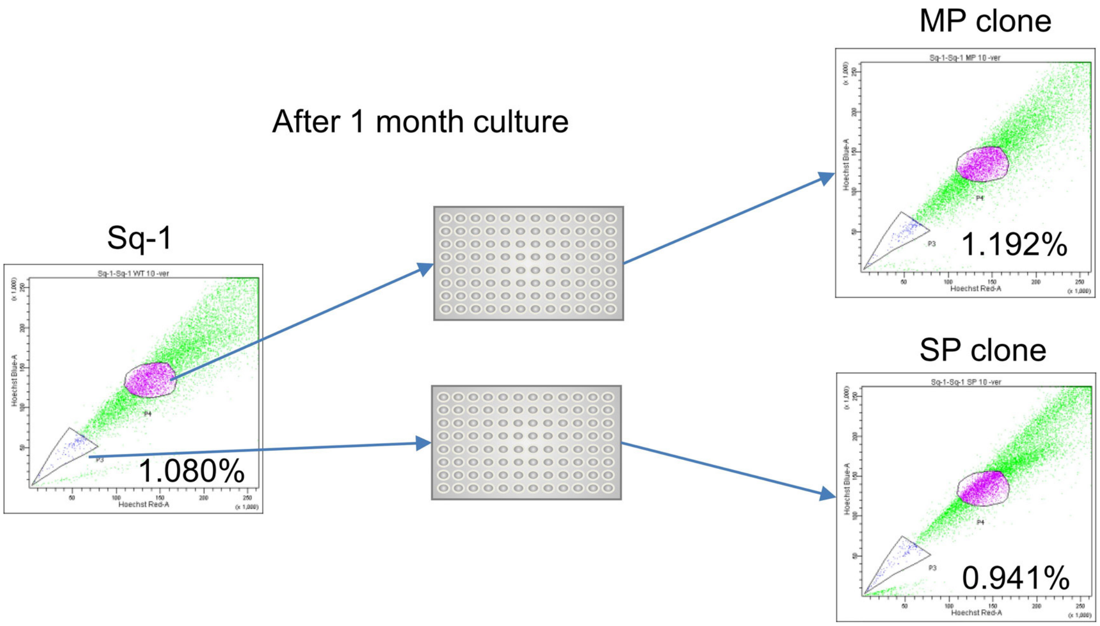

Supplementary Figure S2: Dedifferentiation of lung squamous cell carcinoma cell line, Sq-1.SP analysis revealed Sq-1 cells was positive for SP cells. SP clone and MP clone cells were established by single cell sorting. SP analysis revealed that both SP clone cells and MP clone cells were positive for SP cells. The percentage represents the ratio of SP cells.

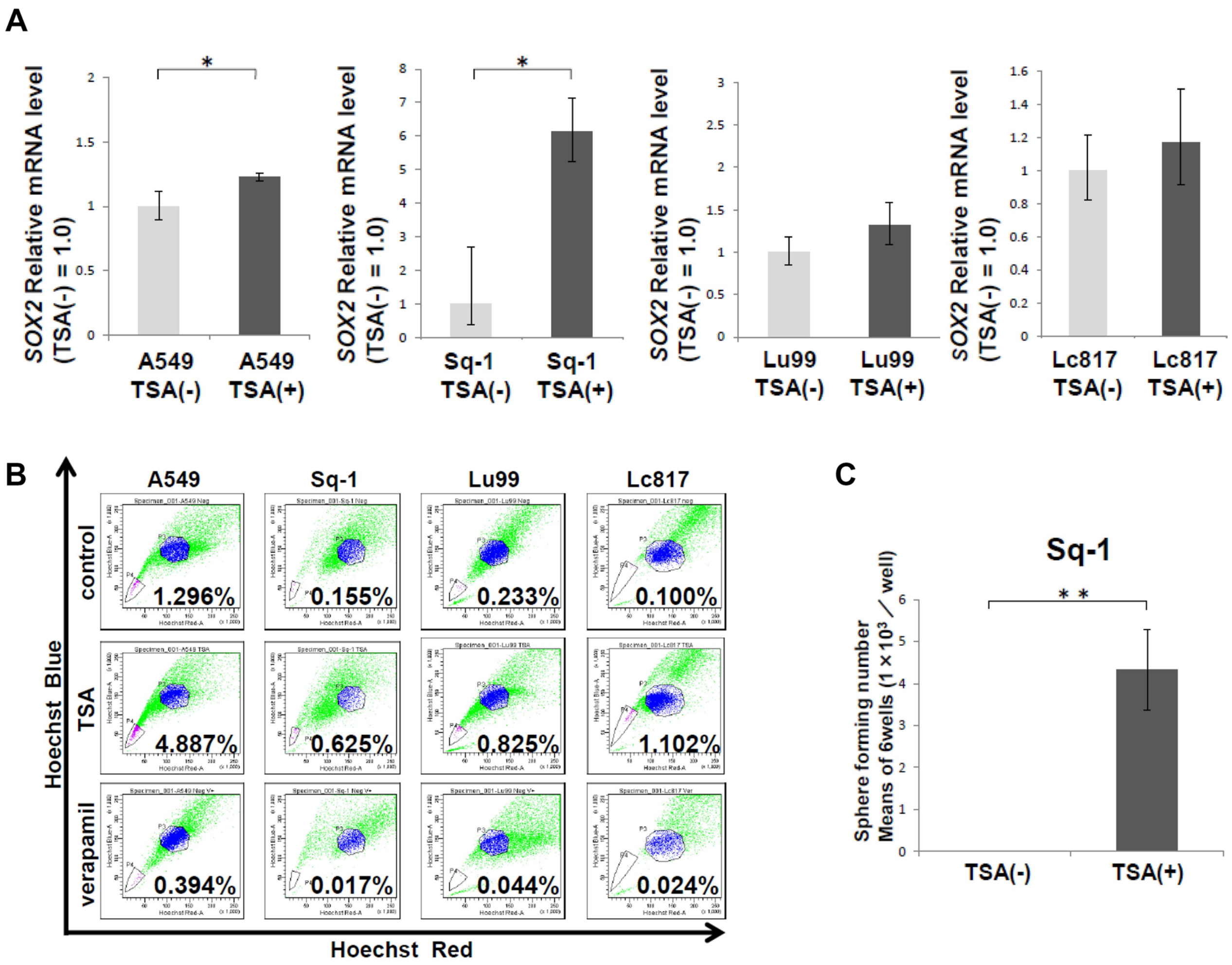

Supplementary Figure S3: *SOX2* expression and stemness were regulated by class I *HDAC* in lung cancer. (**A**) Quantitative real-time PCR analysis of *SOX2* mRNA expression in A549, Sq-1, Lu99 and Lc817 cells treated with TSA. Cells not treated with TSA were used for the control, which was set as 1.0. Data are expressed as means ± s.d. of relative values compared with cells not treated with TSA. Asterisks indicated significant differences. *$P < 0.05$. Paired t-test. (**B**) SP assay of A549, Sq-1, Lu99 and Lc817 cells treated with TSA. The percentage represents the ratio of SP cells. (**C**) Comparison of the numbers of sphere-forming TSA(-) and TSA(+) cells in Sq-1 cells. Asterisks indicated significant differences. **P values. Paired t-test.

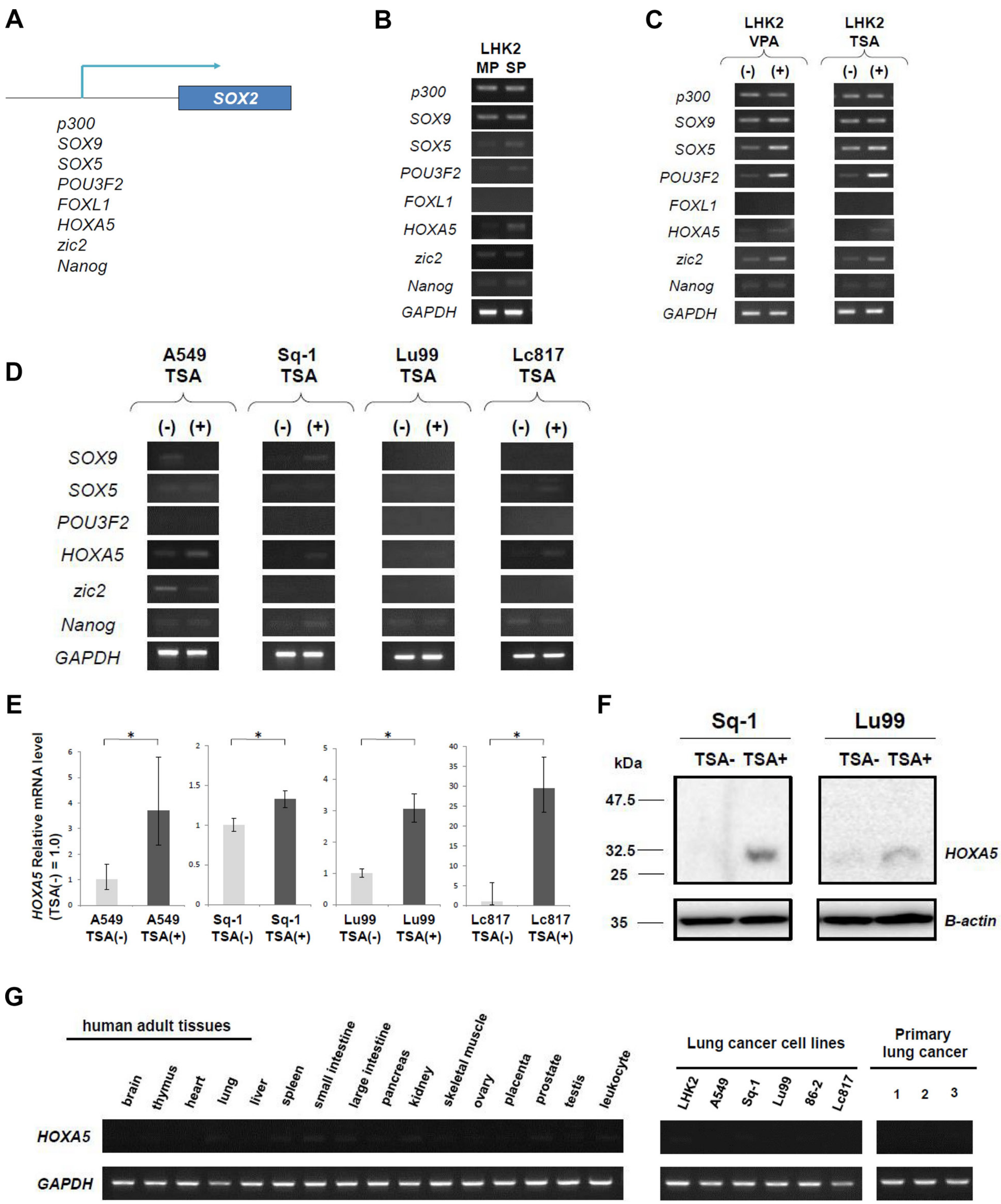
A
SOX2
p300
SOX9
SOX5
POU3F2
FOXL1
HOXA5
zic2
Nanog
B
LHK2
MP SP
GAPDH
C
LHK2 VPA
LHK2 TSA
(-) (+)
D
A549 TSA
Sq-1 TSA
Lu99 TSA
Lc817 TSA
E
HOXA5 Relative mRNA level (TSA(-) = 1.0)
A549 TSA(-)
A549 TSA(+)
Sq-1 TSA(-)
Sq-1 TSA(+)
Lu99 TSA(-)
Lu99 TSA(+)
Lc817 TSA(-)
Lc817 TSA(+)
F
Sq-1
Lu99
kDa
TSA- TSA+
47.5
32.5
25
35
HOXA5
B-actin
G
human adult tissues
brain
thymus
heart
lung
liver
spleen
small intestine
large intestine
pancreas
kidney
skeletal muscle
ovary
placenta
prostate
testis
leukocyte
Lung cancer cell lines
LHK2
A549
Sq-1
Lu99
86-2
Lc817
Primary lung cancer
1 2 3

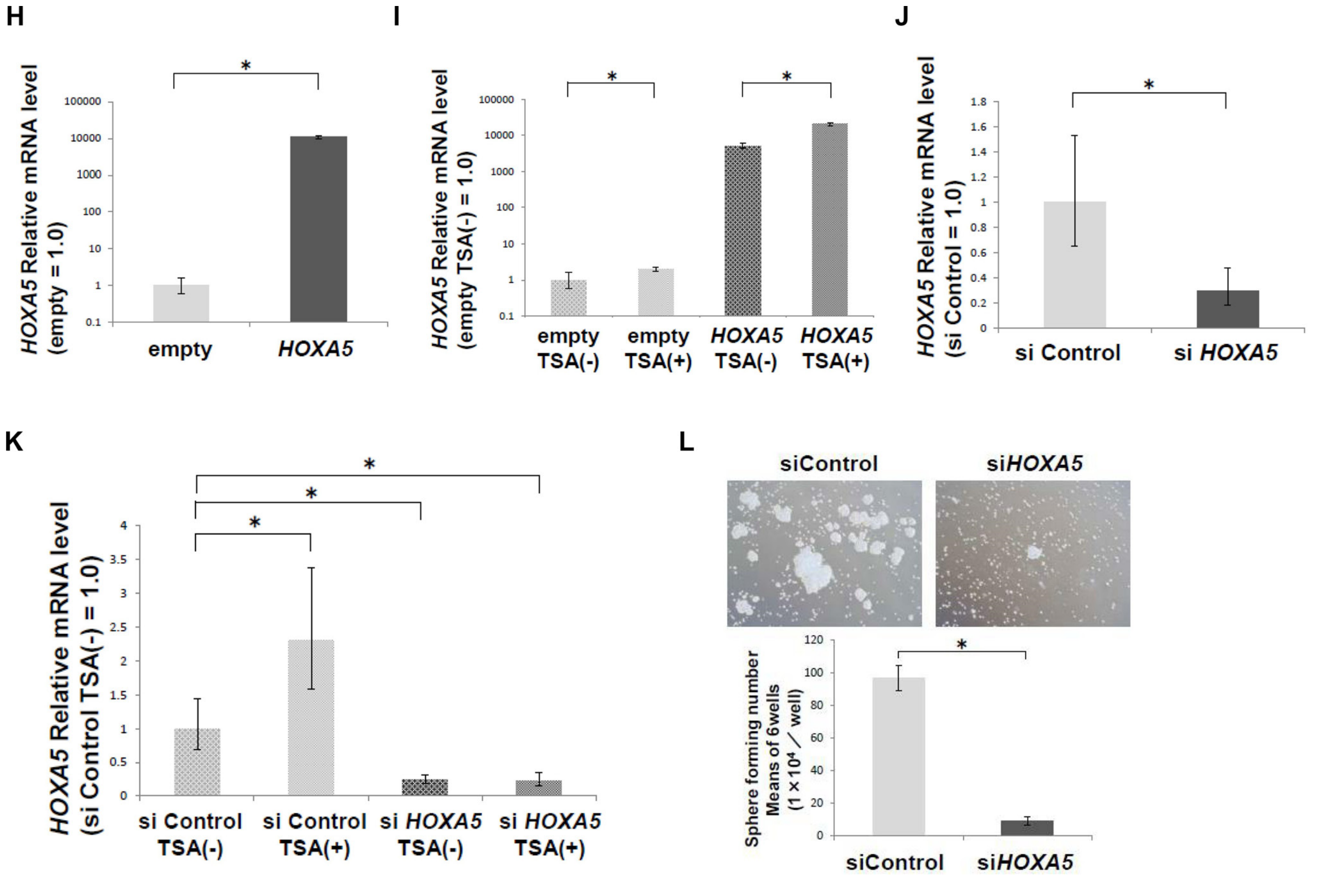

Supplementary Figure S4: ***HOXA5*** **is a candidate transcription factor for** ***SOX2*** **expression and has a role in** ***SOX2*** **expression.** (**A**) A schematic summary of candidate transcription factors that may bind to the *S0X2* promoter area predicted by SABiosciences′ Text Mining Application and UCSC Genome Browser. (**B**) RT-PCR using LHK2 MP and SP cells. Expression of some genes in the *SOX2* promoter area was examined by RT-PCR. *GAPDH* was used as an internal control. (**C**) RT-PCR using LHK2 cells treated with VPA and TSA. Expression of some genes in the *SOX2* promoter area was examined by RT-PCR. *GAPDH* was used as an internal control. (**D**) RT-PCR using A549, Sq-1, Lu99 and Lc817 cells treated with TSA. Expression of some genes in the *SOX2* promoter area was examined by RT-PCR. *GAPDH* was used as an internal control. (**E**) Quantitative real-time PCR analysis of *HOXA5* mRNA expression in A549, Sq-1, Lu99 and Lc817 cells treated with TSA. Asterisks indicated significant differences. *$P < 0.05$. *t*-test. (**F**) Expression of HOXA5 protein in Sq-1 (TSA- and TSA+) cells and Lu99 (TSA- and TSA+) cells confirmed by Western blotting with an anti-HOXA5 antibody. (**G**) RT-PCR using human adult tissues, human lung cancer cell lines and primary lung cancer cells. (**H**) Quantitative real-time PCR analysis of *HOXA5* mRNA expression in LHK2 cells transfected with *HOXA5*. Asterisks indicated significant differences. *$P < 0.05$. *t*-test. (**I**) Quantitative real-time PCR analysis of *HOXA5* mRNA expression in empty vector-transfected TSA(-) cells, empty vector- transfected TSA(+) cells, HOX45-transfected TSA(--) cells and HOX/\5-transfected TSA(+) cells. Asterisks indicated significant differences. *$P < 0.05$. *t*-test. (**J**) Quantitative real-time PCR analysis of *HOXA5* mRNA expression in *HOXA5* siRNA-transfected LHK2 cells. Asterisks indicated significant differences. *$P < 0.05$. Paired *t*-test. (**K**) Quantitative real-time PCR analysis of *HOXA5* mRNA expression in si Control-TSA(-) cells, si Control-TSA(+) cells, si *HOXA5*- TSA(-) cells and si *HOXA5*-TSA(+) cells. Asterisks indicated significant differences. *$P < 0.05$. Paired *t*-test. (**L**) Images of tumor spheres seeded with *HOXA5* siRNA-transfected LHK2 cells. Scale bar, 100 |im. The graph shows a comparison of numbers of sphere-forming Control cells and *HOXA5* siRNA-transfected LHK2 cells. Asterisks indicated significant differences. **P* values, *t*-test.

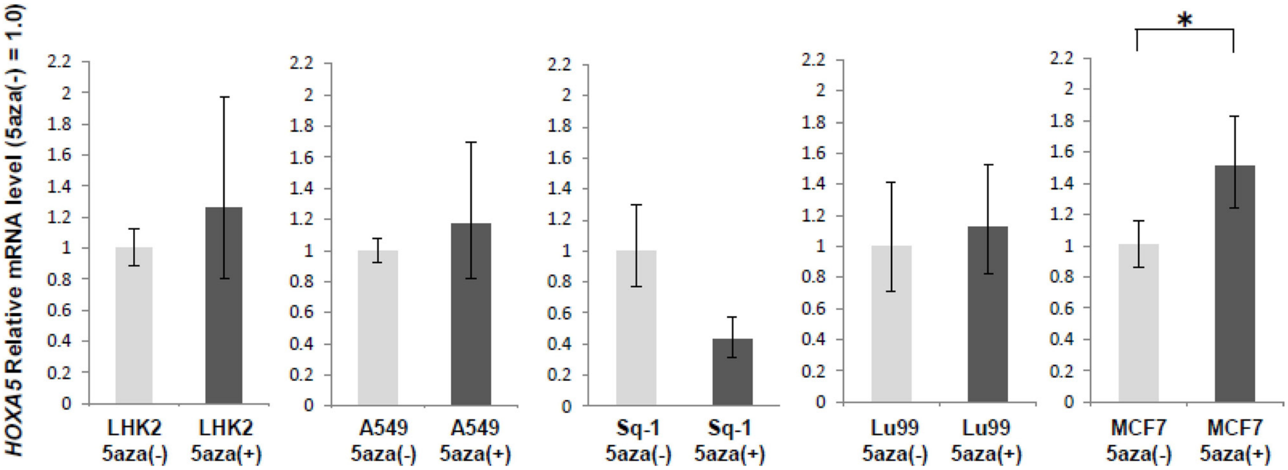

Supplementary Figure S5:De-methylation of *HOXA5* promoter region was not related to lung cancer cells. Quantitative real-time PCR analysis of *HOXA5* mRNA expression in LHK2, A549, Sq-1, Lu99 and MCF7 cells treated with 5aza. Asterisks indicated significant differences *$P < 0.05$. Mest.

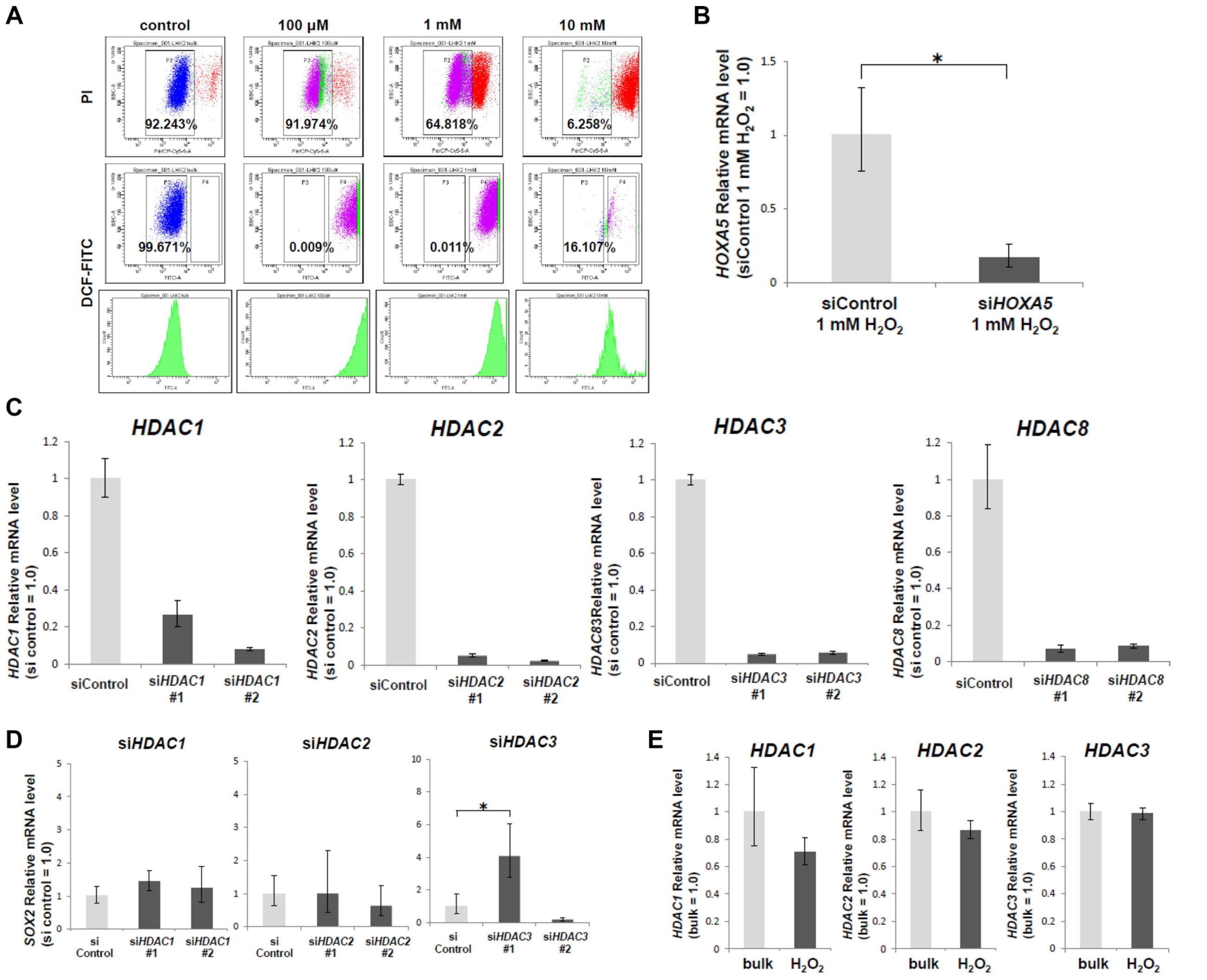

Supplementary Figure S6: Repression of HDAC8 by oxidative stress may be related to acquisition of stemness in lung cancer. (**A**) LHK2 cells were treated with 100 µM, 1 mM and 10 mM H_20_2, and intracellular ROS concentrations were measured by DCF-DA staining. (**B**) Quantitative real-time PCR analysis of *HOXA5* mRNA expression in *HOXA5* siRNA-transfected cells treated with 1 mM H_20_2. Control siRNA-transfected cells treated with 1 mM H_20_2 were used for the control, which was set as 1.0. Data are expressed as means ± s.d. of relative values compared with control cells. Asterisks indicated significant differences. *$P < 0.05$. *t*-test. (**C**) Quantitative real-time PCR analysis of each Class I *HDAC* mRNA expression in each of the Class I *HDAC* siRNA-transfected LHK2 cells. (**D**) Quantitative real-time PCR analysis of *SOX2* mRNA expression in *HDAC1, HDAC2* and *HDAC3* siRNA- transfected LHK2 cells. Asterisks indicated significant differences. *$P < 0.05$. *t*-test. (**E**) Quantitative real-time PCR analysis of *HDAC1, HDAC2* and *HDAC3* mRNA expression in LHK2 cells treated with 1 mM H_20_2.
